# Genetic Demography of the Blue and Red Shrimp, *Aristeus antennatus*: A Female-Based Case Study Integrating Multilocus Genotyping and Morphometric Data

**DOI:** 10.3390/genes13071186

**Published:** 2022-07-01

**Authors:** Alba Abras, Jose-Luis García-Marín, Sandra Heras, Melania Agulló, Manuel Vera, Laia Planella, María Inés Roldán

**Affiliations:** 1 Laboratori d’Ictiologia Genètica, Campus Montilivi, Universitat de Girona, 17003 Girona, Spain; alba.abras@udg.edu (A.A.); joseluis.garcia@udg.edu (J.-L.G.-M.); sandra.heras@udg.edu (S.H.); melania.agullo@udg.edu (M.A.); laia.planella@udg.edu (L.P.); 2 Departamento de Zoología, Genética e Antropología Física, Campus Lugo, Universidad de Santiago de Compostela, 27002 Lugo, Spain; manuel.vera@usc.es

**Keywords:** deep-sea shrimp, genetic demography, horizontal movement, microsatellite loci, recruitment

## Abstract

In this study, we quantified the three key biological processes, growth, recruitment, and dispersal pattern, which are necessary for a better understanding of the population dynamics of the blue and red shrimp *Aristeus antennatus*. This marine exploited crustacean shows sex-related distribution along the water column, being females predominate in the middle slope. The present study attempts to fill the existing gap in the females’ genetic demography, as scarce knowledge is available despite being the most abundant sex in catches. We analyzed morphometric data and genotyped 12 microsatellite loci in 665 *A. antennatus* females collected in two consecutive seasons, winter and summer 2016, at the main Mediterranean fishing ground as a model. Almost every female in summer was inseminated. Five modal groups were observed in both seasons, from 0+ to 4+ in winter and from 1+ to 5+ in summer. Commercial-sized sorting based on fishermen’s experience resulted in a moderate-to-high assertive method concerning cohort determination. Genetic data pointed out females’ horizontal movement between neighboring fishing grounds, explaining the low genetic divergence detected among western Mediterranean grounds. Our results could represent critical information for the future implementation of management measures to ensure long-time conservation of the *A. antennatus* populations.

## 1. Introduction

The blue and red shrimp, *Aristeus antennatus* (*A. antennatus*) (Risso, 1816) (Crustacea, Aristeidae) (En—Blue and red shrimp; Fr—Crevette rouge; Sp—Gamba roja) is the eurybathic species that achieves the broadest vertical distribution in the Mediterranean Sea with reported records from 80 down to around 3000 m depth [1,2]. However, the species shows a demographic structure along the water column. The proportion of males and juveniles tends to increase with depth, whereas females are more abundant in the middle slope [3,4], with peaks of abundance around 700 m [1]. The mating activity starts from late winter to late spring (January–May) when adults of both sexes aggregate at 400–900 m depth, and after mating, males return to deeper waters [4,5]. Although females’ maturity begins in spring, they can store sperm to fertilize the eggs in the absence of males [5,6]. Hence, during the spawning season, shoals are composed mainly of mature adult females [4,7,8]. Then, in autumn and winter, there is a decrease in population density in the middle slope as reproductive shoals break up [9].

Larval dispersal by marine currents during the *A. antennatus* pelagic stage has been pointed out as the main responsible for the high level of genetic connectivity found across the Mediterranean Sea [10,11,12,13,14,15,16,17]. However, Guijarro et al. [18] reported a possible migration of juveniles and adults, and Agulló et al. [16,17] suggested adult migration as a contributor to the high genetic homogeneity found in the western Mediterranean populations. Genetic relatedness analyses showed that spermatophores attached to females collected at 400–700 m depth during the reproductive period came only partially from males captured simultaneously with the females, whereas a large proportion of spermatophores belonged to unsampled and less related males, likely from other spawning grounds [19]. Using genetic assignment analyses, Abras et al. [20] recently reported the horizontal dispersal of juvenile males. On the other hand, females’ horizontal displacements have only been proposed by a single tagging study [21] and, despite their relevant contribution to the fisheries of this species, there are no genetic studies inferring the demography and dispersal of *A. antennatus* females.

At depths shallower than 1000 m, where *A. antennatus* fishery takes place, the species shows the highest abundance with a strong sex-biased ratio to females [4,22]. In the lowest status (>1500 m), where there is no fishing activity (virgin grounds), a sex ratio of 1:1 is reached [9,23]. Furthermore, peaks of juveniles were observed in March between 600 and 1000 m in depth [24], indicating the migration of juveniles from the bottom areas to the mid-slope and their subsequent incorporation into the fishery, which leads to the recovery of *A. antennatus* fishing stocks [2,25,26].

Fishermen sort by size *A. antennatus* individuals into commercial categories on board, resulting in four sizes: small (>70 pieces/Kg), medium (50–70 pieces/Kg), large (30–49 pieces/Kg), and extra-large (<30 pieces/Kg). The average price ranks from 24.14 euros/Kg for small shrimps to 83.03 euros/Kg for the extra-large ones, but seasonal fluctuations are common according to catches and market demand [27]. The small size category is composed of males and females, whereas larger size groups are only represented by females [28]. Females are longer lived (with a life expectancy of 5/6 years) [25,29] and are the most pressured by fishing, especially in summer [4,7]. Although age estimations in this species are mostly based on body sizes (carapace length, e.g., [23,30,31,32]), there is no clear relationship between the aforementioned market sizes and cohorts [33]. Furthermore, the reduced growth rate for older individuals makes age determination difficult due to possible size overlapping between cohorts [34].

The blue and red shrimp fishery along the western Mediterranean coast constitutes a resource of high economic value [35]. However, to date, only a single regional management plan designed for *A. antennatus* exists at Palamós fishing ground (Spain, northwestern Mediterranean Sea). This plan includes meshing size regulations and a temporary cessation of trawling for a period of sixty working days per year [36,37]. Palamós is also one of the most important fishing harbors for *A. antennatus* of the western Mediterranean Sea and has its own quality brand [38]. For all these reasons, this fishing ground has been the focus of several studies based on the species [7,19,20,28,39,40].

In the present study, morphometric and genetic analyses have been integrated to investigate demographical aspects such as growth, recruitment, and horizontal displacements of *A. antennatus* females using the Palamós fishing ground as a model. This approach took advantage of previous genetic information in the same fishing ground on the breeding behavior of the species [19] and on the growth, recruitment, and geographical origin of *A. antennatus* males [20]. The results reported here fill the existing gap in the genetic demography of females, which is the most relevant sex in *A. antennatus* fishery [28].

## 2. Materials and Methods

### 2.1. Biological Material

A total of 665 *A. antennatus* females were collected on board the *Nova Gasela* commercial trawling vessel at Palamós fishing ground (41°54′04″ N, 3°16′08″ E) in two sampling campaigns, one on 3 March (winter) and the other on 7 July (summer) 2016. A single haul per campaign was conducted at 500 m depth, with an average cruising speed of 2 knots and an effective towing duration of 2 h. The codend had a mesh size of 40 mm stretched and was covered by a codend cover with a mesh size of 12 mm stretched (see Abras et al. [20] for further details). As the codend cover is prohibited for commercial hauls, it was specifically authorized for these two scientific campaigns.

Immediately after being captured, the crew sorted by eye all the individuals caught in the codend into four commercial sizes (small, medium, large and extra-large) according to their expertise. A random sample of around 100 adult females from each size group was kept for subsequent analyses, but due to limited availability, all captured females were retained for the largest sizes (Table 1). Females were identified through the presence of thelycum (morphological criteria [33]). In addition, one or more spermatophores adhered to the thelycum were detected in summer individuals, as July corresponds to the peak of the spawning season [19]. In winter, females in the codend cover were identified on board by members of the scientific team according to the morphological criteria. The presence of transparent gonads suggested they were juvenile females, and a sample of around 100 was randomly kept for analysis. In summer, there were no females captured in the codend cover. All kept individuals of each campaign were immediately stored on ice on board and quickly transported to the laboratory. Once there, the carapace length (CL) was measured from the orbital margin to the mid posterior edge of the carapace using a digital vernier caliper, and a piece of approximately 10 mg of the muscle of each individual was preserved in 70% ethanol and stored at room temperature for further genetic analyses.

### 2.2. DNA Extraction and Molecular Markers

Genomic DNA was extracted from the muscle tissue of each female following the phenol-chloroform protocol adjusted to *A. antennatus* by Fernández et al. [41]. Twelve polymorphic microsatellite loci developed for this species were amplified by means of three multiplex PCRs and one PCR singleplex: *Aa123*, *Aa1255*, *Aa138*, *Aa1444*, *Aa496b*, *Aa667*, *Aa681*, *Aa751*, *Aa956*, *Aa1061*, *Aa1195*, and *Aa818* [19,42]. PCR products were analyzed in an ABI PRISM 3730 × l sequencer (Applied Biosystems, Foster City, CA, USA) at the Sequencing Unit of the *Universidade de Santiago de Compostela* (Campus Lugo, Lugo, Spain). Genotyping was carried out using GeneMapper 4.0 software with GeneScan^TM^ 500 LIZ^®®^ size standard (Applied Biosystems).

### 2.3. Size Frequency Distributions, Cohort Identification, and Comparison with Commercial Categories

The CL-frequency distributions in 1 mm class intervals of the females in winter and summer were studied through a modal progression analysis (MPA) implemented in the FISAT II software [43]. The Bhattacharya method [44] was used for the visual identification of the different normal distributed size groups in the polymodal CL-frequency distributions. For each season, results were displayed as the computed mean of each size group with its standard deviation (SD) and a separation index (SI). The latter should be ≥2 between consecutive size groups to assume each one as a single age group; otherwise, it means substantial overlap between cohorts. Specific cohorts in our samples (0+, 1+, 2+, 3+, 4+, and 5+) were assigned, combining MPA results with CL-cohort relationships previously reported for western Mediterranean *A. antennatus* populations.

A one-way ANOVA and Scheffe post-hoc tests included in the IBM SPSS Statistics version 25 package (Armonk, NY, USA) were performed to compare the mean CL in winter and summer of each size group present in both campaigns (small, medium, large, and extra-large). The same analysis was performed for the MPA-identified groups shared in the two sampling seasons (1+, 2+, 3+, and 4+). Cohen’s kappa coefficient (K) [45], which described the level of concordance among methods relating to the observed agreement and the agreement expected by chance, was calculated between the groups of females identified by the MPA and the categories derived from the commercial criteria, including juveniles, in each season. The interpretation of K values was as follows: 0 to 0.20 indicates slight agreement, 0.21 to 0.40 fair agreement, 0.41 to 0.60 moderate agreement or good agreement beyond chance, 0.61 to 0.80 substantial agreement, and 0.81 to 1 almost perfect agreement [45]. Calculations were performed with VassarStats, which is available online at http://www.vassarstats.net/kappa.html (accessed on 23 May 2022).

### 2.4. Genetic Analysis

Genetic diversity at each female commercial size group (including winter juveniles caught in the codend cover) as well as at each age group was estimated as the number of alleles per locus (*N_A_*) and allelic richness (*A_R_*) calculated using FSTAT 2.9.3 [46], and observed (*H_O_)* and expected (*H_E_*) heterozygosities using GENEPOP 4.7.0 [47]. Deviations from Hardy-Weinberg equilibrium (HWE) were summarized using the inbreeding coefficient *F_IS_* and tested using the exact probability test of Guo & Thompson [48], implemented in GENEPOP 4.7.0. Bonferroni correction was used to adjust significance levels. Null allele frequency for each locus was assessed using Micro-Checker 2.2.3 [49]. Genetic distinctions between female commercial size groups and also between age groups in winter and summer were assessed by pairwise *F_ST_* using the program FSTAT 2.9.3 [46].

As previously done with *A. antennatus* males in Abras et al. [20], the parental contribution of 2015 local spawners to the 2016 juvenile and small female groups were analyzed using assignment tests. Putative parental individuals from 2015 included adult females (F; N = 52; mean CL = 38.27 ± 3.76 mm), sympatric males captured with females (M; N = 61; mean CL = 22.78 ± 2.59 mm), and mating males inferred from spermatophores attached to females’ thelycum (S; N = 59) reported by Planella et al. [19]. HYBRIDLAB 1.0 software [50] was used to generate independent F1 offsprings from these 2015 parent genotypes involving mating between females and males (F × M F1) and between females and spermatophore genotypes (F × S F1). Ten sets of paired baselines of 100 simulated offspring individuals from the resulting females and spermatophores (F × S F1 baseline) and 100 individuals from the admixture of females and males (F × M F1 baseline) were used in subsequent assignations. Thus, 25 more independent sets of 100 individuals for each combination (F × S F1 and F × M F1) were also simulated and independently assigned to each of the 10 baseline sets, producing a total of 250 replicates to evaluate the accuracy of the assignments to simulated baselines. Thereafter, the 2016 female groups (winter juveniles, winter small, and summer small) were assigned to each of the 10 baselines. Assignments were carried out using the Bayesian Rannala and Mountain method [51], as implemented in GENECLASS 2 [52]. Individuals with an assignment probability < 0.01 to both baselines were considered as having another source. Comparisons of the results of these latter assignations with those from simulated data were performed using one-way ANOVA and Scheffe post-hoc tests included in IBM SPSS Statistics.

**Figure 1:**
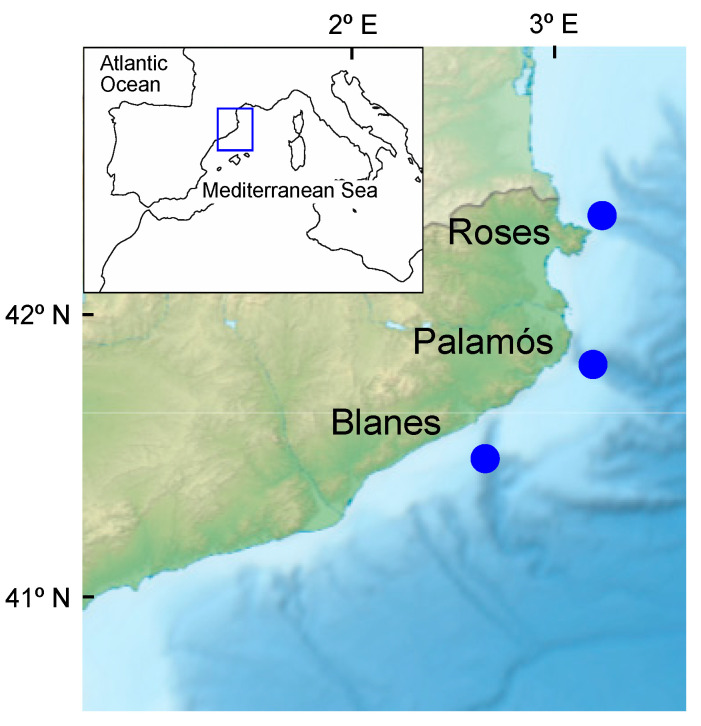
Sampling locations of *A. antennatus*. Relief map: Sémhur/Wikimedia Commons.

In addition, the geographical origin of juvenile and small females was analyzed through assignment tests. Winter juveniles and small females were used as a baseline together with two sets of small females collected during the same winter of 2016 at two nearby fishing grounds: one from Roses (Cap de Creus Canyon, n = 55) situated to the north of Palamós, and the second from Blanes (Blanes Canyon, n = 54), located to the south (Figure 1) [20]. Chi-square tests using VassarStats were computed to compare the assignment pattern of the summer females to winter baselines. Finally, assignment tests were also used to obtain the distribution to winter baselines of female summer groups up to 3+ derived from the MPA. Fisher exact probability tests were used to compare summer assignment patterns to winter baselines.

## 3. Results

### 3.1. Size-Age Composition of A. antennatus Females

**Figure 2:**
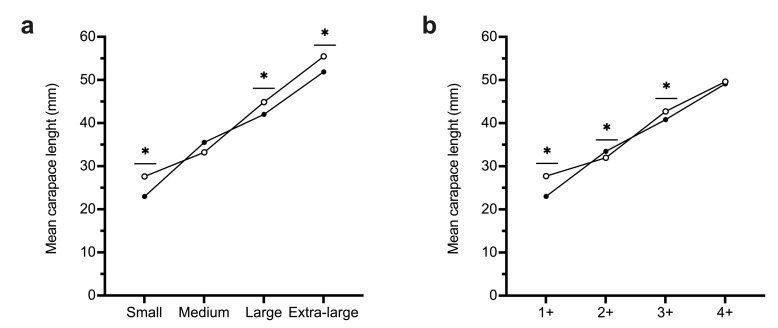
Comparison of the mean carapace length (CL) of *A. antennatus* females based on commercial categories (**a**) and the groups derived from the modal progression analysis (**b**) in winter (filled circles) and summer (empty circles) for each group present in both campaigns. *p* < 0.05 obtained from one-way ANOVA and Post-hoc Scheffe test are indicated with an asterisk (*).

Mean CL was significantly lower in winter than in summer for all commercial female size groups (juveniles from codend cover removed) but the medium-sized category (Figure 2a). Practically all the females caught in summer were inseminated (331/334; 99.1%) and a small proportion of them (27/331; 8.2%) presented more than one spermatophore (Table 1).

**Figure 3:**
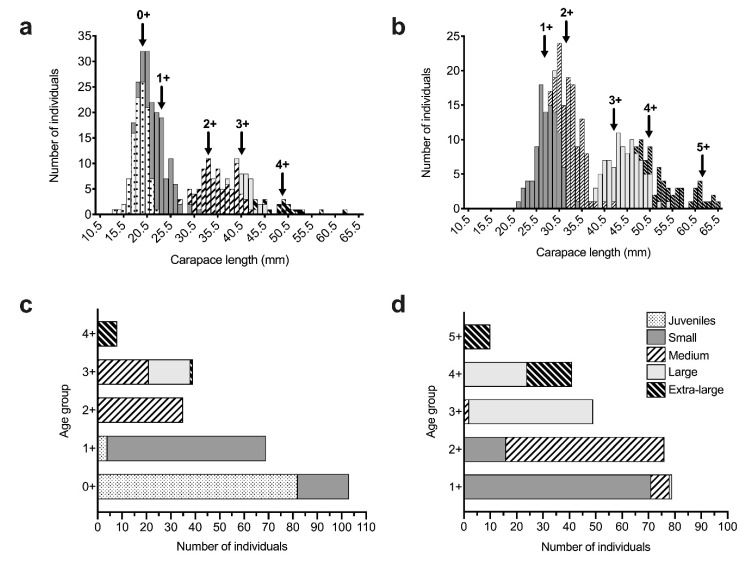
Size frequency distributions (carapace length, CL) of *A. antennatus* female groups in winter (**a**) and in summer (**b**) coupled with commercial size composition of each group derived from the modal progression analysis (MPA) in winter (**c**) and summer (**d**). Arrows indicate the CL mean computed for each age group by the MPA.

The MPA from CL-frequency distributions showed that females in both winter and summer were composed of five separated (SI > 2) modal groups (Table 2, Figure 3a,b). Based on CL-age keys reported in previous studies on *A. antennatus* western Mediterranean fisheries [23,30,31,32,34,53,54] (Table 3), the age of each MPA group was assigned to cohorts from 0+ to 4+ in winter and from 1+ to 5+ in summer. The size of the first identified cohort (19.26 ± 1.77 mm) was slightly larger than the one reported by Carbonell [34] and looks similar to the smaller modal cohort indicated by D’Onghia et al. [23] for samples at the Ionian Sea collected in April and May. Though these latter authors did not use codend cover, but a stretched mesh of 20 mm in the codend and reduced proportions of collected females were in the range between 16 and 26 mm of CL, especially in April catches.

The comparison of the mean CL in winter and summer of each age group present in both campaigns (1+, 2+, 3+, and 4+) resulted in lower values in winter than in summer for all age groups except for the cohort 2+ but only significant in cohorts 1+ and 3+. In the case of the age group 2+ occurred the opposite; the mean CL was significantly higher in winter than in summer (Figure 2b).

**Figure 4:**
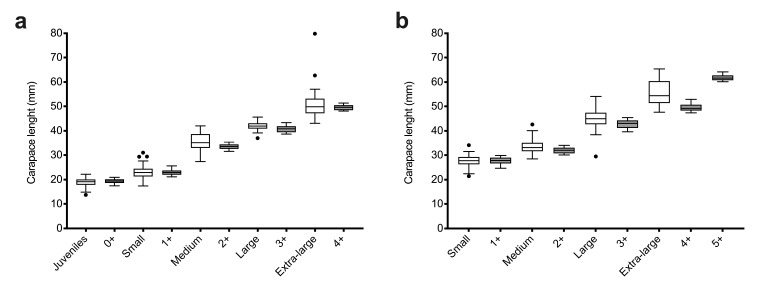
Carapace length (CL) of *A. antennatus* female groups based on commercial categories (white boxes) and the modal progression analysis components (MPA, grey boxes) in winter (**a**) and summer (**b**). The line that cuts through the box indicates the median value.

All females in the range of mean ± SD of each modal group was clearly considered as a member of the cohort and retained to have better baselines for individuals to make comparisons between cohorts and commercial sizes. Using this strategy, 77 out of 331 females captured in winter (23.3%) and 79 out of 334 of those caught in summer (23.7%) remained doubtful and were removed from cohort groups (Table 2). The agreement between these cohort baselines and the commercial sizes was substantial according to Cohen’s kappa either in winter samples (K = 0.75; 95% CI = 0.68–0.81) or in summer ones (K = 0.73; 95% CI = 0.67–0.80) (Table S1). Thus, each cohort baseline was mainly composed of females assigned to a single commercial size by the crew vessel, and only cohorts 3+ in winter and 4+ in summer were clear admixtures between two commercial size groups (Figure 3c,d). The results indicated that the medium commercial size in winter is composed of individuals from the 2+ and 3+ cohorts, and the large and extra-large summer females are also admixtures of two consecutive cohorts (Figure 3a,b). The presence of cohort 0+ females in the small size group retained by the codend in winter indicated that a portion of these young individuals are captured even in a codend of a mesh size of 40 mm. The distribution of CL between each commercial category and the more related MPA group in winter is fairly coincident except for the extra-large females in summer that showed a mean CL value between the two older cohorts (Figure 4).

### 3.2. Genetic Diversity and Genetic Divergence of A. antennatus Females

A total of 665 *A. antennatus* females were genotyped for 12 microsatellite loci (Table S2). Only locus *Aa818* was ruled out from all subsequent analyses due to a null allele frequency > 0.2 in four commercial categories (Table S2) and six age groups (Table S3).

Regarding commercial categories (Table 1), females with medium size reached the maximum mean *A_R_* (5.34, Table 1) among winter samples; meanwhile, the small size group displayed the highest *H_O_* and *H_E_* (0.492 and 0.644, respectively). The females from the large and extra-large size groups showed the lowest diversity values and were the only ones displaying significant departures from HWE (*F_IS_* = 0.284 and 0.303, respectively, Table 1). In summer, diversity indexes were fairly similar to winter ones, but small females achieved the highest value of *A_R_* (5.40) and large females showed the highest *H_O_* and *H_E_* (0.511 and 0.633, respectively). A significant departure from HWE was observed in the small, large, and extra-large females in summer (Table 1). These deviations were related to positive *F_IS_* values, indicating a deficit of heterozygosity, and affect commercial sizes composed of more than a cohort (Figure 3b).

In relation to age groups (Table 2), in winter, the highest mean *A_R_* and *H_E_* were achieved by group 2+ and the maximum *H_O_* by group 1+. Significant departures from HWE genotype expectations were observed in groups 1+ and 4+. In summer, group 5+ showed the maximum result of *A_R_* and *H_E_*_,_ whereas the highest *H_O_* was achieved by group 3+. Significant departures from HWE were observed in groups 3+ and 4+.

Pairwise *F_ST_* between all-female commercial categories as well as age groups were low, ranging from 0.0000 to 0.0047 (Table 4). After Bonferroni correction, *F_ST_* values between paired samples resulted in no significant differences.

### 3.3. Origin of Juvenile and Small Females

**Figure 5:**
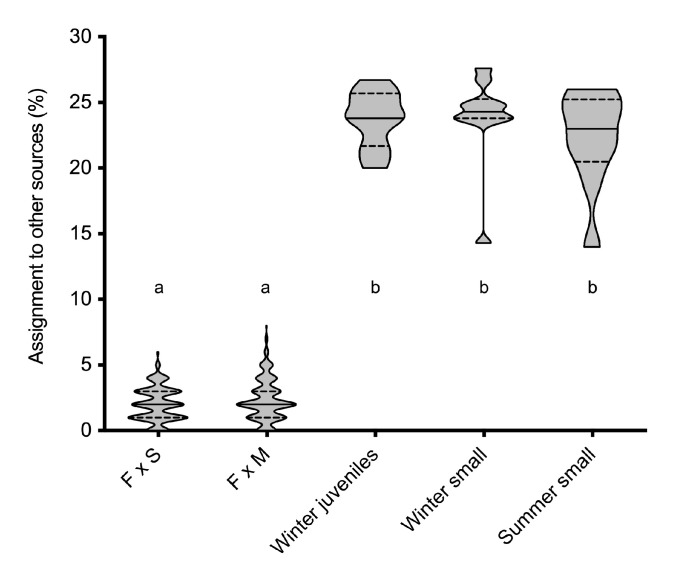
Distribution of the proportion (%) of assignment to other sources of the simulated offspring sets and the 2016 *A. antennatus* female groups. Continuous lines indicate the median value of each group and dashed lines represent the quartiles. Letters (a, b) indicate groups identified by the post-hoc Scheffe test. F, females; S, spermatophores; M, males.

In the assignment tests for the assessment of the parental contribution of 2015 local spawners to the 2016 juvenile and small female groups, the simulated individuals indicated a limited accuracy of baselines (Table 5), probably due to the low but significant divergence between males and spermatophores [19]. However, in both cases, around 60% of the simulated F1 individuals were assigned to the correct baseline (64.5% for F × S; 58.6% for F × M). Winter juvenile females captured in the codend cover and small winter and summer females showed similar assignment patterns showing a higher percentage of assignation to the F × S F1 baseline than to the F × M F1 one. The pattern for capturing females was also distinct from simulated F1 offsprings from local spawners in the larger proportion (>20%) of individuals assigned to other sources (Table 5 and Figure 5).

**Figure 6:**
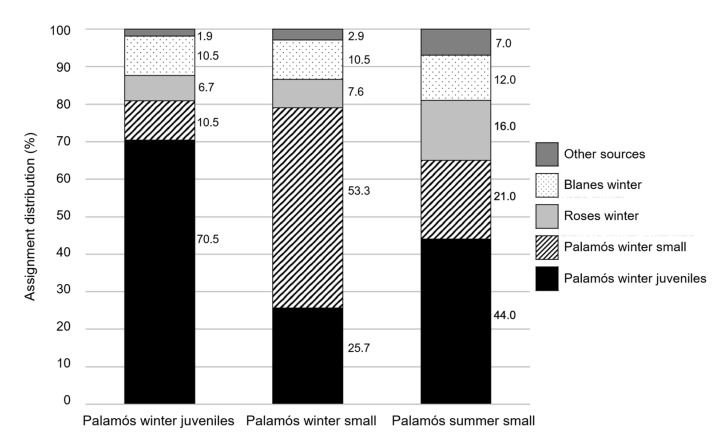
Assignment distribution (%) of *A. antennatus* female commercial categories collected during the summer at Palamós fishing ground.

Assignment tests to analyze the geographical origin of juvenile and small females showed a limited accuracy of the baselines, especially in the case of the small females from Palamós collected in winter (Figure 6). Even so, more than 50% of the females in this group were correctly assigned to their baseline. As previously observed in males [20], the recruitment from other grounds was confirmed by the high proportion of individuals assigned to Roses, Blanes, and other sources, reaching 35% of the total assignments in summer small females. Small summer females showed an assignment pattern significantly different from winter juveniles (*p* = 0.02) and winter small (*p* < 0.0001) but more related to winter juveniles (44% of individuals assigned to this baseline) (Figure 6).

**Figure 7:**
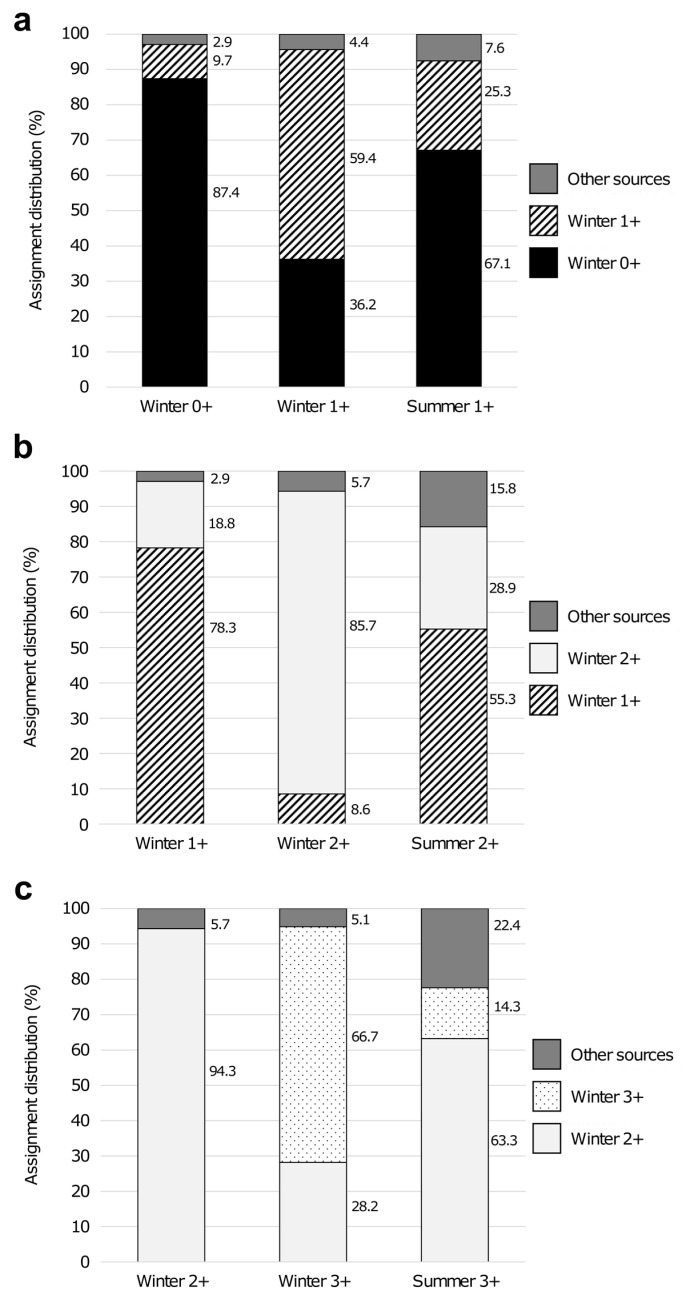
Assignment distribution (%) of *A. antennatus* female summer groups up to 3+ derived from the modal progression analysis (MPA) to their closest winter baselines: summer 1+ (**a**), summer 2+ (**b**) and summer 3+ (**c**).

Finally, the assignment distribution of female MPA groups up to 3+ to their closest winter baselines showed that, although summer cohorts had assignment patterns significantly different from winter baselines in all cases (Fisher exact test *p* < 0.005), they were assigned in a higher proportion to their predecessor year cohort in winter (Figure 7). However, summer cohorts had a percentage of contribution of their same year cohort in winter (from 14.3 in summer females 3+ to 28.9% in summer females 2+) and also input from other sources (up to 22.4% in summer females 3+) (Figure 7).

## 4. Discussion

### 4.1. Size-Age Composition and Commercial Categories of A. antennatus Females

As reported by Demestre & Lleonart [25], females and males should be analyzed separately because they have different demographic patterns: (i) Females and males show ontogenic differential distribution [55]. (ii) Females live longer than males [25]. (iii) Females are more abundant in catches [25]. (iv) The size of the catches is larger in females, indicating the size dimorphism of the species [25,56], which is consistent with both the present study and the previous one focused on males carried out during the same sampling campaigns [20]. Small females were bigger (mean CL = 25.26 ± 3.39 mm) than males in the same sampling (mean CL = 21.92 ± 2.83 mm) [20] (*t*-test *p* < 0.001). (v) Females have greater age diversity and size composition [55]. (vi) Both sexes become sexually active within the first year of their life [5,56], but females show seasonal maturity from May to October, with a peak in July–August, whereas males are mature during the whole year [33]. Size at first maturity (CL_50_) can be influenced by environmental factors and fluctuate between geographical areas [55]. Sardà & Demestre [33] reported a CL_50_ of 27 mm in females, with some mature females from 23 mm in the Catalan Sea. Demestre & Martín [57] calculated a CL_50_ of 26 mm in the same area. Carbonell et al. [55] also reported a CL_50_ of 26 mm in females in the Balearic Sea whereas García-Rodríguez & Esteban [56] reported a CL_50_ of 21.9 mm in the same place. In this study, the mean CL in the small size category was 22.96 mm in winter and 27.65 mm in summer, with most females inseminated (Table 1), being consistent with the aforementioned studies.

Related to the MPA analysis (Table 2), the number of estimated age groups and the size of each group was quite similar to data reported by other studies conducted close to our area of study (Table 3), i.e., Gulf of Alicante, Gulf of Vera and Balearic Islands, [34,53,54]. However, our data do not differ substantially from other studies conducted in more remote areas either, i.e., Ligurian, Tyrrhenian, and Ionian Seas [23,30,31,32]. Indeed, the largest size-age ratio differences between groups were observed in older age groups, approximately from 4+ onwards, in which it becomes difficult to distinguish the cohorts due to age-related reduction of growth rate [34]. These differences could be explained by several reasons: (i) Oceanographic conditions are different in the areas analyzed with a broad range of variables exerting influence, such as climate, hydrodynamics, trophic resources, and predators [18,31]. The species must adapt to the different hydrological conditions and environmental factors [1,58]. (ii) The lack of an appropriate experimental method to estimate growth parameters. Although other methods have been proposed for age determination, such as the quantification of the age-pigment lipofuscin or tagging studies [32,54], further investigation is needed to prove their efficiency. Most studies make age estimations based exclusively on sizes, so results could be slightly biased by the influence of molting activity [59], fishing habits [31], and the exploitation pattern, which brings increased pressure on females [34]. (iii) The widespread recruitment throughout the Mediterranean Sea. Growth taxes could differ between individuals that recruit on the lower slope (slower growth) and the ones doing it in shallower waters (faster growth). Thus, they could not be distinguished when joining since individuals of the same age would reach slightly different sizes [23]. Data reported by Carbonell [34] estimated a monthly growth rate of 2 mm for juveniles and 1 mm for adults. Nevertheless, analysis of growth in crustaceans is challenging because it is a discontinuous process carried out by molting [60], so the comparison between studies and areas is extremely difficult and not always feasible.

*A. antennatus* individuals are sorted after fishing by the fisherman according to commercial categories, which are not specifically based on quantitative criteria and do not depend on cohorts. On the other hand, the MPA allows an objective way of sorting, but it is not easy to identify the different groups in the polymodal CL-frequency distributions, especially in the largest sizes corresponding to the oldest cohorts. Both methods reached a substantial agreement (K = 0.75 and 0.73 in winter and summer, respectively) being almost all age groups mainly represented by a single commercial category (contribution between 79% and 100%) with the exception of cohorts 3+ in winter and 4+ in summer, thus pointing out fishermen’s sorting based on years of experience as a generally assertive method. However, the pooling of different cohorts into the same commercial category also took place, as observed in the medium size in winter and the large and extra-large categories in summer (Figure 3).

### 4.2. Recruitment and Geographical Origin of A. antennatus Females

Genetic data pointed out for the first time that mating males consisted of males sympatric with females and also males from other spawning grounds [19]. Furthermore, a study based exclusively on *A. antennatus* males detected that males sampled at fishing grounds during the spawning season have a limited contribution to the spermatophores attached to females [20]. That previous study reported through assignment tests the genetic correlation of most males collected in 2016 to the offspring of 2015 spawners with high contribution from other sources. Likewise, genetic analyses in the present work based on the same simulated F1 of individuals collected in 2015 indicate that most juvenile and small females in 2016 were also correlated to the offspring of 2015 spawners with a considerable assignment to other sources (Table 5 and Figure 5). Thus, our results confirmed a displacement of males, either vertical movement inside Palamós fishing ground or horizontal displacement from adjacent fishing grounds, as previously suggested [19,20].

Since Blanes and Roses females from winter 2016 were used in the assignment tests to analyze the geographical origin of juvenile and small females had small size (mean CL of 24.23 and 27.61 mm for Blanes and Roses, respectively) [16], only juvenile and small females in the present study could be assigned. Winter juvenile females had a higher contribution than the small ones to summer small females indicating that this latter group was mainly composed of juvenile females that have grown and females that have moved from other grounds (Figure 6). These results were confirmed by the assignment distribution of female MPA groups up to 3+ to their closest winter baselines (Figure 7). In all cases, summer female groups were assigned by over 55% to their predecessor year cohort in winter, indicating again the growth of the winter individuals to join the next summer cohort group. However, the same year cohort in winter, as well as females from other grounds, also contributed to the group in summer, as indicated by the percentage of assignation to these sources. Data reported by Abras et al. [20] in males pointed out a limited contribution of winter male juveniles to the summer pre-adult male groups, thus differing from results on females (present study). Otherwise, in both sexes, the level of contribution from adjacent fishing grounds is high. More than 40% of males in summer groups originated from outside the Palamós ground [20], whereas the percentage of contribution from neighboring grounds to summer small females rose to 35% (present study) (Figure 6). Our results corroborate the connectivity between nearby fishing grounds of *A. antennatus* in the north-western Mediterranean Sea, which likely occurs through the dispersal of different stages of the life-cycle of *A. antennatus*, i.e., larvae, juveniles, and adults of both sexes [16,17,20,40].

The dispersal of individuals from one ground to another involves the combination of residents and immigrants, which could lead to the low genetic differentiation (*F_ST_*) observed with no significant variation in either commercial categories or age groups (Table 4). On the other hand, Cannas et al. [13] hypothesized that *A. antennatus* females could disperse more easily than males due to its main presence in intermediate currents that flow faster than deep currents. Recently, Agulló et al. [17] reported the existence of horizontal displacements of *A. antennatus* females and a high genetic connectivity between spawning females’ grounds within the Mediterranean Subarea GSA6.

## 5. Conclusions

Combining morphometric and genetic data, this study provides new insights into *A. antennatus* growth, recruitment, and mobility pattern with particular emphasis on females. Commercial-sized sorting based on fishermen’s experience allows a fast classification of *A. antennatus* captures according to market indications, which indirectly results in a moderate-to-high assertive method concerning females’ cohort determination. Although, it should be kept in mind that there could be individuals belonging to more than one cohort in a commercial category. The present genetic data detail the horizontal movement of *A. antennatus* females between neighboring fishing grounds, which is in agreement with the high genetic connectivity pattern detected for *A. antennatus* populations in the north-western Mediterranean Sea. Our findings could help to improve the current protection measures and ensure the long-term sustainability of the fishery.

## Figures and Tables

**Table 1:** Commercial size groups of *Aristeus antennatus* (*A. antennatus*) females, including sampling season, commercial category, mean carapace length (CL) with standard deviation (SD), number of females (N), number of females with one or more spermatophores adhered to the thelycum, and estimates of genetic diversity (excluding locus *Aa818*).

Sampling Season	Commercial Category	Mean CL ± SD (mm)	N	Females with Spermatophore (Double)	*N_A_*	*A_R_*	*H_O_*	*H_E_*	*F_IS_*
Winter	Juvenile ^1^	18.98 ± 1.53	105	-	9.7	5.32	0.46	0.63	0.27
	Small	22.96 ± 2.52	105	-	8.9	5.24	0.49	0.64	0.23
	Medium	35.54 ± 3.41	82	-	9.2	5.34	0.43	0.64	0.33
	Large	42.03 ± 1.97	22	-	5.7	4.64	0.43	0.60	0.28 *
	Extra-large	51.86 ± 8.65	17	-	5.7	5.07	0.42	0.61	0.30 *
Summer	Small	27.65 ± 2.37	100	99	9.7	5.40	0.51	0.63	0.19 *
	Medium	33.24 ± 2.46	99	97 (3)	9.4	5.39	0.48	0.62	0.22
	Large	44.88 ± 3.78	91	91 (14)	9.4	5.36	0.51	0.63	0.19 *
	Extra-large	55.46 ± 5.10	44	44 (9 + 1 triple)	7.8	5.14	0.45	0.62	0.27 *

^1^ Juvenile is not a commercial category. *N_A_*, Number of alleles; *A_R_*, allelic richness; *H_O_*, observed heterozygosity; *H_E_*, expected heterozygosity; *F_IS_*, inbreeding coefficient. * Significant departure from Hardy-Weinberg equilibrium after Bonferroni correction (α/99, *p* < 0.0005).

**Table 2:** Description of the groups identified through the modal progression analysis of the carapace length (CL) frequency distributions of *A. antennatus* females, including sampling season, modal and age group, mean carapace length with standard deviation (SD), separation index (SI), number of females (N), and estimates of genetic diversity (excluding locus *Aa818*).

Sampling Season	Modal Group	Age Group	Mean CL ± SD (mm)	SI	N	*N_A_*	*A_R_*	*H_O_*	*H_E_*	*F_IS_*
Winter	1	0+	19.26 ± 1.77	-	103	9.7	3.94	0.46	0.64	0.28
	2	1+	23.38 ± 2.23	2.06	69	8.3	3.88	0.49	0.64	0.24 *
	3	2+	33.51 ± 2.08	4.70	35	7.0	3.95	0.45	0.65	0.31
	4	3+	40.89 ± 2.54	3.19	39	7.2	3.71	0.41	0.63	0.34
	5	4+	49.12 ± 2.41	3.33	8	4.3	3.75	0.43	0.64	0.33 *
Summer	1	1+	27.26 ± 2.72	-	79	9.3	3.88	0.51	0.63	0.19
	2	2+	32.06 ± 1.97	2.05	76	9.2	3.95	0.51	0.63	0.20
	3	3+	42.53 ± 2.94	4.26	49	7.9	3.90	0.52	0.64	0.19 *
	4	4+	50.22 ± 2.97	2.60	41	7.7	3.83	0.47	0.62	0.24 *
	5	5+	62.09 ± 2.26	4.54	10	5.2	3.98	0.45	0.64	0.30

*N_A_*, Number of alleles; *A_R_*, allelic richness; *H_O_*, observed heterozygosity; *H_E_*, expected heterozygosity; *F_IS_*, inbreeding coefficient. * Significant departure from Hardy-Weinberg equilibrium after Bonferroni correction (α/110, *p* < 0.0005).

**Table 3:** Comparison of the age groups of *A. antennatus* females with previous studies on Mediterranean populations.

Reference	Geographic Region	Sampling Period	Cohort						
			1	2	3	4	5	6	7
Present study	Palamós (Spain, north-western Mediterranean Sea)	Winter 2016	19.26 ± 1.77	23.38 ± 2.23	33.51 ± 2.08	40.89 ± 2.54	49.12 ± 2.41	-	-
		Summer 2016	-	27.26 ± 2.72	32.06 ± 1.97	42.53 ± 2.94	50.22 ± 2.97	62.09 ± 2.26	
García-Rodríguez (2003) [53]	Gulf of Alicante (Spain, western Mediterranean Sea)	January 1995–December 1998	-	25.65	33.66	43.19	51.16	-	-
	Ibiza Channel (Spain, western Mediterranean Sea)	January 1992–December 1994	-	29.18	37.56	45.75	54.1	-	-
	Gulf of Vera (Spain, western Mediterranean Sea)	January 1992–December 1994	-	29.1	-	43.8	52.6	-	-
Carbonell (2005) [34]	Balearic Islands and other Mediterranean Sea regions and Atlantic Ocean data compilation	12 years	14–16	20–25	30	38–40	48–50	55–58	>60
Orsi-Relini et al. (2013) ^1^ [32]	Ligurian Sea (western Mediterranean Sea)	1994–2004	-	19–29	32–38	40–45	47–50	52	54
Vila-Gordillo (2001) [54]	Alborán Sea (Spain, western Mediterranean Sea)	June 1997	-	23.88 ± 2.38	29.15 ± 2.06	33.58 ± 2.32	39.20 ± 2.65	47.42 ± 5.04	-
Arculeo et al. (2011) [31]	San Vito Lo Capo (Sicily, Italy, western Mediterranean Sea)	June 2006–May 2007	-	25.25	36.19	46.44	57	-	-
	Terrasini (Sicily, Italy, western Mediterranean Sea)	June 2006–May 2007	-	23.91	32.29	42.86	51.85	-	-
D’Onghia et al. (2009) [23]	North-western Ionian Sea (eastern Mediterranean Sea)	April 2006	-	17.00 ± 1.49	30.96 ± 2.28	35.88 ± 1.81	48.35 ± 2.96	-	-
		May 2006	-	18.16 ± 2.59	31.00 ± 2.39	39.71 ± 2.85	45.69 ± 2.56	52.37 ± 2.06	-
		June 2006	-	20.79 ± 2.63	30.05 ± 1.50	-	42.10 ± 4.45	51.43 ± 1.60	-
		September 2006	-	24.35 ± 2.71	-	-	38.39 ± 2.33	49.63 ± 2.85	-
Papaconstantinou and Kapiris (2001) [30]	Greek Ionian Sea (eastern Mediterranean Sea)	January–December 1997	-	30.0	-	41.0	49.0	54.0	-

^1^ Data corresponds to the pooled data (MEDITS plus GRUND trawl surveys).

**Table 4:** Pairwise *F_ST_* among *A. antennatus* female groups (below diagonal) and *p*-values (above diagonal).

**Commercial Categories**											
	**Win Juv**	**Win S**	**Win M**	**Win L**	**Win XL**		**Sum S**	**Sum M**	**Sum L**	**Sum XL**	
**Win Juv**	-	0.1600	0.8300	0.2100	0.4200	**Sum S**	-	0.7750	0.1167	0.9083	
**Win S**	0.0030	-	0.9350	0.0150	0.6650	**Sum M**	0.0000	-	0.2583	0.7083	
**Win M**	0.0008	0.0000	-	0.5300	0.9250	**Sum L**	0.0039	0.0015	-	0.4583	
**Win L**	0.0018	0.0003	0.0000	-	0.5050	**Sum XL**	0.0000	0.0000	0.0000	-	
**Win XL**	0.0047	0.0000	0.0000	0.0000	-						
**Modal Progression Analysis Groups**											
	**Win 0+**	**Win 1+**	**Win 2+**	**Win 3+**	**Win 4+**		**Sum 1+**	**Sum 2+**	**Sum 3+**	**Sum 4+**	**Sum 5+**
**Win 0+**	-	0.3250	0.6100	0.3150	0.3250	**Sum 1+**	-	0.9250	0.0700	0.9650	0.8350
**Win 1+**	0.0023	-	0.3250	0.0400	0.3200	**Sum 2+**	0.0000	-	0.5270	1.0000	0.4250
**Win 2+**	0.0000	0.0000	-	0.0850	0.6950	**Sum 3+**	0.0000	0.0000	-	0.8400	0.5450
**Win 3+**	0.0000	0.0017	0.0000	-	0.6800	**Sum 4+**	0.0000	0.0000	0.0000	-	0.3900
**Win 4+**	0.0000	0.0000	0.0000		-	**Sum 5+**	0.0000	0.0000	0.0000	0.0000	-

Win, winter; Sum, summer; J, juveniles; S, small; M, medium; L, large; XL, extra-large. No significant deviations after Bonferroni correction (α/10, *p* < 0.005 for modal progression analysis groups and winter commercial categories and α/6, *p* < 0.0083 for summer commercial categories) were observed.

**Table 5:** Assignment distribution (%) and standard deviation of *A. antennatus* female commercial categories to simulated offspring from 2015 spawner genotypes.

	Replicates	F × S F1 Baseline	F × M F1 Baseline	Other Sources
**F x S F1**	250	64.5 ± 8.4	33.5 ± 8.3	2.0 ± 1.3
**F x M F1**	250	39.1 ± 8.2	58.6 ± 8.4	2.3 ± 1.6
**Winter juveniles**	10	39.5 ± 8.2	36.8 ± 9.3	23.7 ± 4.5
**Winter small**	10	41.8 ± 10.7	34.4 ± 12.5	23.8 ± 3.6
**Summer small**	10	40.4 ± 9.0	37.3 ± 10.1	22.3 ± 3.7
**All categories**	10	40.6 ± 9.3	36.1 ± 10.2	23.3 ± 3.2

F, females; S, spermatophores; M, males.

## Data Availability

Not applicable.

## Supplementary Materials

The following supporting information can be downloaded at: https://www.mdpi.com/article/10.3390/genes13071186/s1, Table S1: Concordances used to calculate Cohen’s kappa coefficient (K) for the comparison between commercial categories and modal progression analysis (MPA) groups in winter and summer; Table S2: Genetic diversity for twelve microsatellite loci for commercial-sized *A. antennatus* female groups; Table S3: Genetic diversity for twelve microsatellite loci for *A. antennatus* female groups identified through the modal progression analysis of the carapace length (CL) frequency distributions.
