# Supplementary material for: Genetic Demography of the Blue and Red Shrimp, Aristeus antennatus: A Female-Based Case Study Integrating Multilocus Genotyping and Morphometric Data
Source: Genes (Basel). 2022 Jul 1;13(7):1186. doi: 10.3390/genes13071186 (PMC9320127; doi:10.3390/genes13071186)
Supplement: Supplementary file 1 [file genes-13-01186-s001.zip › genes-1766796-supplementary.pdf]

Supplementary information for

Genetic demography of the blue and red shrimp, *Aristeus antennatus*: A female-based case study integrating multilocus genotyping and morphometric data

Alba ABRAS,[1] José-Luis GARCÍA-MARÍN,[1] Sandra HERAS,[1] Melania AGULLÓ,[1] Manuel VERA,[2] Laia PLANELLA[1] and María Inés ROLDÁN[1]

[1] Laboratori d'Ictiologia Genètica, Universitat de Girona, Girona, Spain.

[2] Departamento de Zoología, Genética y Antropología Física, Universidad de Santiago de Compostela, Lugo, Spain.

Correspondence: María Inés Roldán, Laboratori d'Ictiologia Genètica, Universitat de Girona, Campus Montilivi, E-17003 Girona, Spain. Email: marina.roldan@udg.edu

This pdf file includes:

Supplementary Table S1

Supplementary Table S2

Supplementary Table S3

Table S1. Concordances used to calculate Cohen's kappa coefficient (K) for the comparison between commercial categories and modal progression analysis (MPA) groups in winter and in summer.

Winter

		Commercial categories						Total
		J	S	M	L	XL	NC	
MPA groups	0+	82	21	0	0	0	0	103
	1+	4	65	0	0	0	0	69
	2+	0	0	35	0	0	0	35
	3+	0	0	21	17	1	0	39
	4+	0	0	0	0	8	0	8
	NC	19	19	26	5	8	0	77
Total		105	105	82	22	17	0	331

Summer

		Commercial categories					Total
		S	M	L	XL	NC	
MPA groups	1+	71	7	1	0	0	79
	2+	16	60	0	0	0	76
	3+	0	2	47	0	0	49
	4+ and 5+	0	0	24	27	0	51
	NC	13	30	19	17	0	79
Total		100	99	91	44	0	334

J, juveniles; S, small; M, medium; L, large; XL, extra-large; NC, not classified by MPA.

Table S2 Genetic diversity for twelve microsatellite loci for commercial sized *Aristeus antennatus* female groups.

Group		*Aa123*	*Aa1255*	*Aa138*	*Aa1444*	*Aa496b*	*Aa667*	*Aa681*	*Aa751*	*Aa956*	*Aa1061*	*Aa1195*	*Aa818*
Winter juveniles	*n*	104	102	104	100	99	103	105	101	101	105	105	105
	N_A	5	15	22	14	2	7	20	3	6	8	5	6
	A_R	3.386	7.276	11.051	6.995	1.951	3.911	9.051	2.009	4.931	4.504	3.424	4.690
	H_O	0.4231	0.4804	0.7981	0.4500	0.2222	0.4175	0.6000	0.0990	0.6040	0.3429	0.5810	0.3429
	H_E	0.6155	0.7128	0.9095	0.7502	0.2141	0.6905	0.8127	0.1806	0.7100	0.7047	0.6049	0.6726
	F_{IS}	0.3126*	0.3261*	0.1225*	0.4001*	-0.0380	0.3954*	0.2617*	0.4519*	0.1493	0.5135*	0.0396	0.4902*
	Nu	0.1195	0.1412	0.0576	0.1701		0.1598	0.1114	0.0745	0.0546	0.2320		0.2036
Winter small	*n*	103	101	104	101	101	95	104	97	103	104	104	103
	N_A	6	15	19	11	2	8	18	3	6	6	4	6
	A_R	3.569	7.964	10.388	6.025	1.948	4.628	9.074	2.094	4.425	4.296	3.178	4.410
	H_O	0.4757	0.4554	0.8654	0.4851	0.2376	0.4842	0.6250	0.1753	0.5243	0.4327	0.6154	0.3883
	H_E	0.6147	0.8055	0.8968	0.7545	0.2103	0.7160	0.8193	0.2720	0.6157	0.6744	0.6242	0.6394
	F_{IS}	0.2261	0.4346*	0.0351	0.3570*	-0.1299	0.3237*	0.2371*	0.3557	0.1485	0.3584*	0.0141	0.3926*
	Nu	0.0944	0.1744		0.1391		0.1405	0.0854	0.0687	0.0576	0.1185		0.1461
Winter medium	*n*	79	80	80	79	79	75	81	76	79	82	82	80
	N_A	5	13	20	11	2	9	19	3	6	8	5	6
	A_R	3.572	7.316	10.637	5.830	1.952	4.836	9.120	2.602	4.624	4.716	3.497	4.515
	H_O	0.3924	0.4000	0.8125	0.4051	0.2405	0.4400	0.5432	0.0789	0.5190	0.3049	0.6098	0.3250
	H_E	0.5873	0.7743	0.8972	0.7318	0.2128	0.7046	0.8193	0.3417	0.6716	0.6790	0.6268	0.6458
	F_{IS}	0.3319*	0.4834*	0.0944	0.4465*	-0.1304	0.3755*	0.3370*	0.7689*	0.2273	0.5510*	0.0271	0.4968*
	Nu	0.118	0.2128		0.1829		0.1382	0.1266	0.2048	0.0877	0.2225		0.2145
Winter large	*n*	17	16	22	22	20	17	22	18	14	21	22	22
	N_A	3	7	13	6	2	3	12	3	5	5	4	5
	A_R	2.989	5.881	8.998	5.137	1.804	3.000	8.111	2.610	4.745	4.338	3.446	4.700
	H_O	0.4118	0.1875	0.7727	0.5909	0.1000	0.2941	0.5455	0.0556	0.5000	0.3333	0.7273	0.0909
	H_E	0.5919	0.6354	0.8344	0.7208	0.0974	0.6176	0.7673	0.3399	0.6429	0.6381	0.6093	0.7294
	F_{IS}	0.3043	0.7049*	0.0739	0.1802	-0.0270	0.5238	0.2891	0.8365	0.2222	0.4776	-0.1936	0.8754*
	Nu	0.1743	0.2832		0.1678			0.1477	0.2024				0.3608
	n	12	11	17	17	16	15	17	17	16	17	17	16

Winter extra-large	N_A	4	6	14	6	2	6	11	3	4	4	3	5
	A_R	3.917	6.000	10.786	5.508	1.976	5.338	9.295	2.529	3.975	3.529	2.963	4.901
	H_O	0.2500	0.3636	0.9412	0.5882	0.1875	0.4667	0.3529	0.1765	0.5625	0.2353	0.4706	0.1875
	H_E	0.5909	0.7955	0.8621	0.7445	0.1750	0.6405	0.8989	0.1691	0.6937	0.5974	0.5754	0.7208
	F_{IS}	0.5769	0.5429	-0.0917	0.2099	-0.0714	0.2714	0.6074*	-0.0435	0.1892	0.6062*	0.1821	0.7399*
	Nu							0.3672					0.2841
Summer small	n	100	95	100	98	100	100	100	92	100	99	100	98
	N_A	5	14	22	13	2	8	23	3	6	7	4	6
	A_R	3.587	7.539	11.156	6.770	1.857	4.231	9.229	2.069	4.823	4.966	3.142	4.585
	H_O	0.5000	0.4632	0.8800	0.5408	0.1600	0.5800	0.6700	0.1196	0.6500	0.4343	0.5600	0.3571
	H_E	0.6412	0.7690	0.9058	0.7222	0.1479	0.6875	0.8205	0.2218	0.6941	0.7085	0.5909	0.6104
	F_{IS}	0.2202	0.3977*	0.0284	0.2512	-0.0820	0.1564	0.1834*	0.4610*	0.0635	0.3870*	0.0523	0.4149*
	Nu	0.0993	0.1797		0.0999		0.0634	0.1094	0.0901		0.1692		0.1840
Summer medium	n	99	90	98	95	97	95	98	97	98	99	99	98
	N_A	4	16	20	12	2	8	20	3	6	7	5	6
	A_R	3.103	7.932	10.824	7.019	1.847	4.172	9.019	2.202	4.636	5.079	3.460	4.367
	H_O	0.4343	0.5000	0.8469	0.5053	0.1546	0.4526	0.6939	0.1340	0.5306	0.4141	0.6566	0.3469
	H_E	0.5971	0.8060	0.9078	0.7770	0.1434	0.6992	0.8150	0.1973	0.6192	0.7131	0.5965	0.6178
	F_{IS}	0.2726	0.3796*	0.0670	0.3497*	-0.0787	0.3526*	0.1486	0.3206	0.1431	0.4192*	-0.1007	0.4384*
	Nu	0.0943	0.1668		0.1517		0.1540	0.0506	0.0465	0.0677	0.1720		0.1353
Summer large	n	88	83	88	85	90	86	90	84	88	91	90	89
	N_A	4	15	20	12	2	9	19	3	6	8	5	5
	A_R	3.411	7.340	11.378	7.480	1.953	4.396	8.062	2.187	4.298	4.956	3.521	4.279
	H_O	0.5114	0.5422	0.8523	0.5412	0.2444	0.5116	0.6444	0.1071	0.5114	0.4725	0.6556	0.3371
	H_E	0.6165	0.7898	0.9210	0.8277	0.2156	0.6982	0.7436	0.2235	0.6152	0.6714	0.6096	0.6419
	F_{IS}	0.1706	0.3136*	0.0747*	0.3462*	-0.1338	0.2672	0.1334	0.5205*	0.1688	0.2962	-0.0754	0.4749*
	Nu	0.0788	0.1708	0.0375	0.1516		0.0818	0.0524	0.0968		0.1617		0.1864
Summer extra-large	n	42	36	44	43	42	37	42	42	41	44	43	44
	N_A	5	13	17	11	2	5	16	3	5	6	3	6
	A_R	3.846	7.894	9.971	6.736	1.603	3.956	8.518	2.184	4.059	4.794	2.995	4.960

H_O	0.4286	0.6111	0.7045	0.5581	0.0714	0.4865	0.5714	0.0714	0.4634	0.5000	0.5349	0.2955
H_E	0.6376	0.8163	0.8964	0.7738	0.0697	0.7001	0.8098	0.1974	0.6332	0.7061	0.6030	0.7077
F_{IS}	0.3279	0.2513	0.2140*	0.2787	-0.0250	0.3051	0.2944*	0.6382	0.2682	0.2919	0.1129	0.5825*
Nu	0.1261	0.1003	0.1099	0.0889		0.1096	0.0788			0.1224		0.1990

n, number of genotyped females; N_A, Number of alleles; A_R, allelic richness; H_O, observed heterozygosity; H_E, expected heterozygosity; F_{IS}, inbreeding coefficient; *Nu*, null allele frequency. * Significant departure from Hardy-Weinberg equilibrium after Bonferroni correction (α/108, $P < 0.0005$).

Table S3. Genetic diversity for twelve microsatellite loci for *Aristeus antennatus* female groups identified through the modal progression analysis of the cephalothorax length (CL) frequency distributions.

Group		*Aa123*	*Aa1255*	*Aa138*	*Aa1444*	*Aa496b*	*Aa667*	*Aa681*	*Aa751*	*Aa956*	*Aa1061*	*Aa1195*	*Aa818*
Winter 0+	*n*	102	100	102	99	98	100	103	98	100	103	103	103
	N_A	5	15	21	13	2	8	21	3	6	8	5	6
	A_R	3.053	5.086	6.997	4.855	1.705	3.460	5.894	1.756	3.929	3.705	2.891	3.762
	H_O	0.4808	0.4500	0.8137	0.5051	0.2041	0.3800	0.6019	0.1327	0.6000	0.3592	0.5243	0.3398
	H_E	0.6071	0.7677	0.9114	0.7721	0.2003	0.6895	0.8229	0.2098	0.7051	0.7038	0.5973	0.6877
	F_{IS}	0.2087	0.4138*	0.1072	0.3458*	-0.0189	0.4489*	0.2685*	0.3676	0.1491	0.4896*	0.1222	0.5058*
	Nu	0.0882	0.1751	0.0507	0.1513		0.1674	0.1090	0.0722	0.0596	0.2085		0.1952
Winter 1+	*n*	68	67	68	65	66	62	68	63	67	68	68	67
	N_A	5	12	18	11	2	7	18	3	6	5	4	6
	A_R	3.067	5.021	6.960	4.684	1.739	3.940	5.723	1.914	3.475	3.136	3.005	3.471
	H_O	0.4118	0.4627	0.8971	0.4923	0.2424	0.5484	0.5582	0.1429	0.5224	0.3824	0.6471	0.4328
	H_E	0.6358	0.7732	0.9078	0.7722	0.2145	0.7398	0.8201	0.2837	0.5925	0.6411	0.6340	0.6140
	F_{IS}	0.3523	0.4016*	0.0118	0.3625*	-0.1304	0.2588	0.2828*	0.4964*	0.1183	0.4036*	-0.0206	0.2950
	Nu	0.1413	0.1419		0.1322		0.1248	0.1128	0.0879		0.1290		0.1177
Winter 2+	*n*	33	34	35	34	35	31	35	34	34	35	35	35
	N_A	4	9	16	7	2	7	14	3	6	6	3	6
	A_R	3.108	4.849	6.936	4.704	1.810	3.791	5.716	2.412	3.752	3.543	2.829	3.614
	H_O	0.3939	0.4118	0.8286	0.3529	0.2857	0.4516	0.5714	0.0588	0.5882	0.4286	0.5714	0.2571
	H_E	0.5975	0.7741	0.9063	0.8030	0.2479	0.7177	0.8193	0.3944	0.6430	0.6689	0.5866	0.6458
	F_{IS}	0.3407	0.4680*	0.0858	0.5605*	-0.1525	0.3708*	0.3026	0.8508*	0.0852	0.3593	0.0258	0.6018*
	Nu	0.1413	0.2175		0.2430		0.1303	0.1006	0.2708		0.1104		0.2525
Winter 3+	*n*	34	33	38	38	37	32	39	32	31	38	39	36
	N_A	3	8	18	7	2	7	16	2	6	6	4	5
	A_R	2.885	4.107	6.785	3.797	1.595	3.770	5.864	1.871	3.916	3.213	2.984	3.788
	H_O	0.3529	0.2121	0.8158	0.5263	0.1622	0.3125	0.5128	0.0312	0.5484	0.2632	0.7179	0.1944
	H_E	0.6368	0.7225	0.8979	0.6650	0.1509	0.7067	0.8185	0.2933	0.7188	0.6394	0.6245	0.7032
	F_{IS}	0.4458	0.7064*	0.0915	0.2086	-0.0746	0.5578*	0.3735*	0.8935*	0.2371	0.5884*	-0.1496	0.7235*

	Nu	0.2087	0.2547				0.2343	0.1639	0.2112		0.2624		0.2451
Winter 4+	*n*	5	6	8	8	8	8	8	8	7	8	8	8
	N_A	4	4	7	6	2	5	7	2	4	3	3	4
	A_R	4.000	3.818	5.339	5.302	1.964	4.089	5.920	1.625	3.647	2.625	2.867	3.742
	H_O	0.4000	0.3333	1.0000	0.6250	0.3750	0.3750	0.3750	0.1250	0.4286	0.3750	0.2500	0.0000
	H_E	0.6750	0.7833	0.7768	0.8661	0.3214	0.6875	0.9196	0.1250	0.7143	0.6071	0.5893	0.7500
	F_{IS}	0.4074	0.5745	-0.2874	0.2784	-0.1667	0.4545	0.5922	0.0000	0.4000	0.3824	0.5758	1.0000*
	Nu							0.4286					0.4286
Summer 1+	*n*	79	74	79	77	79	79	79	74	79	79	79	77
	N_A	5	13	20	13	2	8	21	3	6	7	4	6
	A_R	3.108	5.144	6.891	4.539	1.557	3.469	5.643	1.761	3.841	3.927	2.840	3.523
	H_O	0.5063	0.5000	0.8734	0.5844	0.1519	0.5443	0.6582	0.0946	0.6329	0.4557	0.5316	0.3636
	H_E	0.6365	0.7878	0.9044	0.7294	0.1412	0.6797	0.8020	0.2070	0.6746	0.7175	0.5918	0.6098
	F_{IS}	0.2045	0.3653*	0.0343	0.1988	-0.0759	0.1992	0.1793*	0.5429*	0.0618	0.3649*	0.1016	0.4037*
	Nu	0.1008	0.1696		0.0742		0.0866	0.1003	0.1029		0.1730		0.1734
Summer 2+	*n*	76	70	75	74	75	73	76	73	75	75	76	75
	N_A	3	14	21	12	2	7	21	3	6	7	5	6
	A_R	2.891	4.913	7.183	4.950	1.510	3.504	5.938	1.876	3.735	3.881	3.025	3.368
	H_O	0.4342	0.5143	0.8667	0.5405	0.1333	0.5068	0.6974	0.1644	0.6133	0.4267	0.6711	0.3733
	H_E	0.6265	0.7816	0.9169	0.7652	0.1252	0.7002	0.8348	0.2420	0.6465	0.7114	0.5979	0.6064
	F_{IS}	0.3069	0.3420*	0.0548	0.2936*	-0.0647	0.2762	0.1647	0.3208	0.0513	0.4002*	-0.1224	0.3843*
	Nu	0.0997	0.1465		0.1348		0.1116	0.0769			0.1465		0.1377
Summer 3+	*n*	47	44	48	46	49	46	49	45	47	49	48	49
	N_A	4	13	18	9	2	7	16	2	5	6	5	5
	A_R	3.009	5.248	7.323	4.994	1.747	3.569	5.245	1.712	3.491	3.583	2.936	3.604
	H_O	0.5957	0.5000	0.8958	0.5870	0.2449	0.4348	0.6531	0.0889	0.5532	0.4694	0.6250	0.3469
	H_E	0.6422	0.8158	0.9253	0.8060	0.2168	0.7159	0.7617	0.2010	0.6376	0.6735	0.5962	0.6607
	F_{IS}	0.0724	0.3871*	0.0319	0.2718	-0.1294	0.3927	0.1426	0.5578	0.1324	0.3030*	-0.0483	0.4749*
	Nu		0.1862		0.1062		0.1474		0.1049		0.1881		0.2217
Summer 4+	*n*	39	35	39	37	39	38	39	38	39	41	40	40
	N_A	4	10	17	11	2	7	15	2	6	7	4	6

	A_R	3.001	4.326	7.257	5.582	1.574	3.296	4.958	1.644	3.731	3.728	2.995	3.495
	H_O	0.4872	0.6000	0.8205	0.4595	0.1538	0.4737	0.5641	0.0789	0.4329	0.5122	0.6000	0.2000
	H_E	0.6255	0.7538	0.9261	0.8446	0.1437	0.6547	0.7520	0.1717	0.6852	0.6640	0.6234	0.6506
	F_{IS}	0.2211	0.2040	0.1140	0.4560*	-0.0704	0.2765	0.2499	0.5375	0.3639	0.2287	0.0375	0.6926*
	Nu		0.1164		0.1799						0.1042		0.2117
Summer 5+	*n*	10	8	10	10	9	9	10	9	10	10	10	10
	N_A	4	8	9	7	2	4	9	1	4	6	3	5
	A_R	2.998	6.374	5.981	5.010	1.837	3.840	6.051	1.000	3.467	4.289	2.951	3.940
	H_O	0.2000	0.6250	0.6000	0.7000	0.2222	0.4444	0.6000	0.0000	0.6000	0.3000	0.6000	0.2000
	H_E	0.6222	0.9018	0.8611	0.7944	0.2083	0.7569	0.8667	0.0000	0.7111	0.6556	0.6556	0.7556
	F_{IS}	0.6786	0.3069	0.3032	0.1189	-0.0667	0.4128	0.3077		0.1563	0.5424	0.0847	0.7353*
	Nu			0.1397									0.2222

n, number of genotyped females; N_A, Number of alleles; A_R, allelic richness; H_O, observed heterozygosity; H_E, expected heterozygosity; F_{IS}, inbreeding coefficient; *Nu*, null allele frequency. * Significant departure from Hardy-Weinberg equilibrium after Bonferroni correction (α/120, P < 0.0004).
